# Supplementary figures and images for: The chromatin-remodeling enzyme CHD3 plays a role in embryonic viability but is dispensable for early vascular development
Source: PLoS One. 2020 Jul 13;15(7):e0235799. doi: 10.1371/journal.pone.0235799 (PMC7357745; doi:10.1371/journal.pone.0235799)

**Figure 1E**

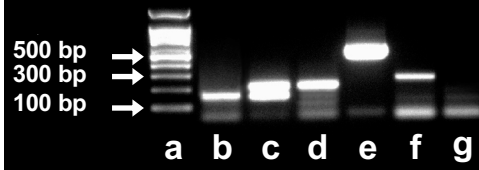

**Figure 4A**

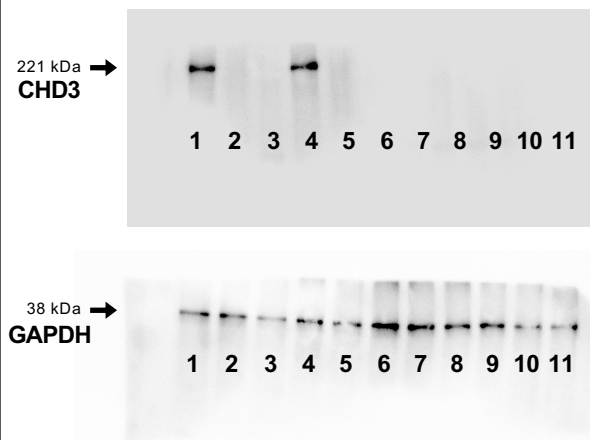

**Figure 4B**

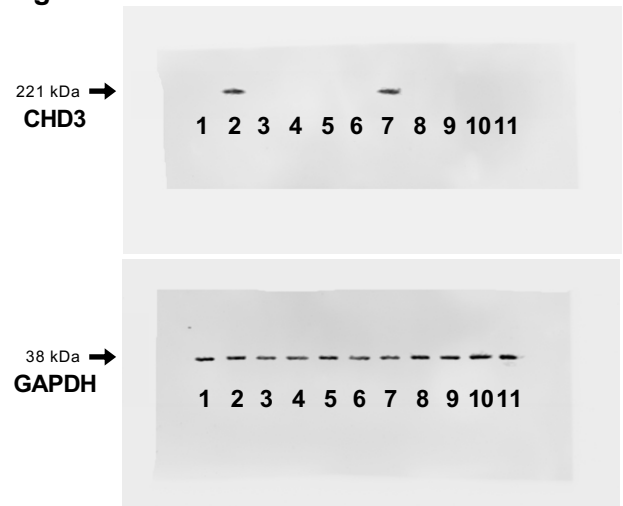

**Figure 5**

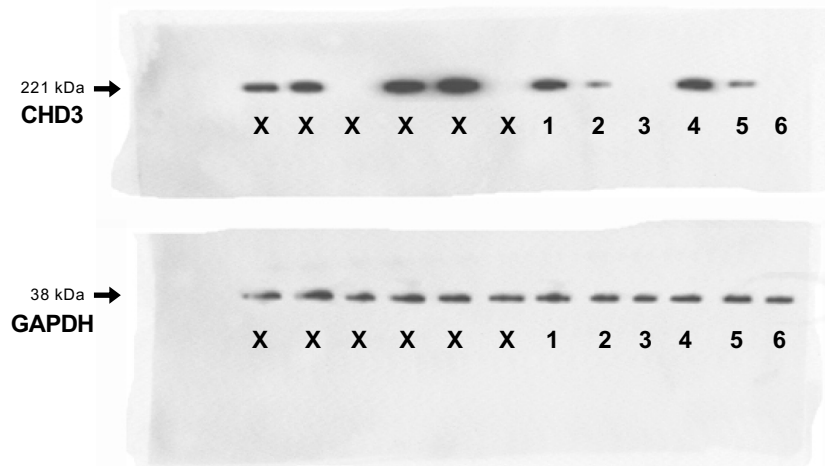

Supplement: S1 Raw images — (PDF) [file pone.0235799.s001.pdf]
