# Supplementary material for: The chromatin-remodeling enzyme CHD3 plays a role in embryonic viability but is dispensable for early vascular development
Source: PLoS One. 2020 Jul 13;15(7):e0235799. doi: 10.1371/journal.pone.0235799 (PMC7357745; doi:10.1371/journal.pone.0235799)
Supplement: Attachment — Submitted filename: Review of PONE-D-20-12024 5-19-2020.pdf [file pone.0235799.s002.pdf]

Review of PONE-D-20-12024, “The Chromatin-remodeling enzyme CHD3 plays a role in embryonic viability but is dispensable for early vascular development.”

In this manuscript, J. Xie and colleagues generated a conditional (floxed) allele of murine CHD3, a component of the NuRD chromatin-remodeling complex. Subsequent analysis of Tie2- and Sox2-Cre-mediated deletion of the *Chd3*-floxed allele revealed no apparent role for CHD3 in embryonic vascular development, as was previously reported for CHD4. Interestingly, a potential requirement for CHD3 activity was suggested by Chd3-knockout mice. Global deletion of CHD3 resulted in partial lethality of mice during gestation, based on observed versus expected $Chd3^{\Delta/\Delta}$ offspring at weaning. Western blot analyses of tissue-specific expression of CHD3 revealed that CHD3 is primarily expressed in adult brain and gonads. Based on these findings, the authors conclude that CHD3 may play a role in embryonic neurodevelopment.

Comments:

1. Although the authors showed that efficient, Cre-mediated deletion of the *Chd3*-flox allele and lack of CHD3 protein in adult $Chd3^{\Delta/\Delta}$ brain tissue, validation of the *Chd3*-flox allele would be augmented by addition of the Southern blot analysis of targeted ES cells and PCR-based analyses of Cre-mediated recombination. The authors list these analyses as “data not shown.” In my opinion, this data should be included in Figure 1.
2. In the discussion section, the authors suggest that “temporal neuronal-specific deletion of Chd3 could further clarify its roles in embryonic and postnatal brain development…,” although no evidence was presented in the manuscript that CHD3 is actually expressed in developing neuronal tissue. Evidence of embryonic, tissue- and developmental stage-specific CHD3 expression would be useful in guiding future studies using *Chd3*-flox mice, and should be included in this manuscript.
